# RCER: Reliable Cluster-based Energy-aware Routing protocol for heterogeneous Wireless Sensor Networks

**DOI:** 10.1371/journal.pone.0222009

**Published:** 2019-09-19

**Authors:** Khalid Haseeb, Naveed Abbas, Muhammad Qaisar Saleem, Osama E. Sheta, Khalid Awan, Naveed Islam, Waheed ur Rehman, Tabinda Salam

**Affiliations:** 1 Department of Computer Science, Islamia College Peshawar, Peshawar, Pakistan; 2 School of Electrical Engineering and Computer Science, National University of Science and Technology, Islamabad, Pakistan; 3 College of Science, Zagazig University, Zagazig, Egypt; 4 Department of Computer Science, Comsats University Islamabad, Attock, Pakistan; 5 Department of Computer Science, University of Peshawar, Peshawar, Pakistan; 6 Department of Computer Science, Shaheed Benazir Bhutto Women University, Peshawar, Pakistan; Northeastern University, CHINA

## Abstract

Nowadays, because of the unpredictable nature of sensor nodes, propagating sensory data raises significant research challenges in Wireless Sensor Networks (WSNs). Recently, different cluster-based solutions are designed for the improvement of network stability and lifetime, however, most of the energy efficient solutions are developed for homogeneous networks, and use only a distance parameter for the data communication. Although, some existing solutions attempted to improve the selection of next-hop based on energy factor, nevertheless, such solutions are unstable and lack a reducing data delivery interruption in overloaded links. The aim of our proposed solution is to develop Reliable Cluster-based Energy-aware Routing (RCER) protocol for heterogeneous WSN, which lengthen network lifetime and decreases routing cost. Our proposed RCER protocol make use of heterogeneity nodes with respect to their energy and comprises of two main phases; firstly, the network field is parted in geographical clusters to make the network more energy-efficient and secondly; RCER attempts optimum routing for improving the next-hop selection by considering residual-energy, hop-count and weighted value of Round Trip Time (RTT) factors. Moreover, based on computing the measurement of wireless links and nodes status, RCER restore routing paths and provides network reliability with improved data delivery performance. Simulation results demonstrate significant development of RCER protocol against their competing solutions.

## 1. Introduction

WSN can be defined as a collection of self-organized minute devices, named as sensor nodes. All deployed sensor nodes are dispersed in a random manner based on ad-hoc infrastructure, to gather the sensory data over the entire network field. Unlike other wireless communication technologies, the WSNs pose unique constraints on the communication protocols because of constrained resources [1]. Routing protocols in traditional networks are designed in a way to improve network performance in terms of data delivery and network latency. On the other hand, WSNs mainly emphasis on how to progress energy preservation while slightest communication overheads [2–4]. Over the conventional networking approaches [5,6], scalability, minor costs, correctness, consistency, and easiness of distribution are focal advantages of WSN applications. Due to limited constraints of in WSN scenarios [7–10], energy utilization is a rare source and has to cope intelligently with improving network lifetime and routing performance. Traditional and single tier routing solutions are not feasible for sensor-based applications, because of the dynamic behavior of sensor nodes. Thus, recently, different researchers [11–13] focus on the development of adaptive and robust routing protocol for the improvement of energy efficiency and appropriate routes discovery towards the endpoints.

Hierarchical-based routing protocols are alternate concepts that are widely used to support efficient route discovery and energy efficiency for WSNs [14–17]. Basically, such schemes are useful in those environments that required scalability to hundreds and thousands of sensor nodes with efficient load distribution. The hierarchical-based network is separated into two foremost components i.e. development of network paneling and data communication. However, the existing hierarchical-based solutions are focused on probabilistic methods and paradigm sub-optimal panels [18,19]. Moreover, route discovery mechanism in existing solutions [17,20–22] are not optimized according to adaptive behavior of wireless communication links and perform periodic re-clustering. In addition, to establish an end-to-end data propagation route, many route request messages are flooded in a hop-by-hop manner, which incurs additional communication cost and reduces network lifetime [23–25]. Accordingly, design and development of energy-efficient and robust routing protocol are needed for energy constraints applications. Furthermore, the optimum selection of routing paths and their re-tuning for data disseminating raises a demanding issue [26–29].

This research paper addresses the problem of scarce energy resources while collecting and forwarding sensory information in WSNs, which shortens network lifetime. Our proposed protocol focuses on developing Reliable Cluster-based Energy-aware Routing for heterogeneous WSNs to increase stability period with the least data relaying interval and route breakages. The proposed protocol firstly parts the sensor nodes into geographically based clusters. Secondly, provides a light-weight solution to optimize the route detection process in terms of hop-count, residual energy, and RTT factors. In addition, the routing paths are updated based on network measurements for supporting network reliability. This may lead to a decrease in end-to-end delay and energy consumption with high data delivery performance.

The research article is structured as follows. The several energy-aware routing schemes in the context of WSN and detailed motivation of the research paper are discussed in Section 2. Section 3 presents the limitations of the existing solution with problem definition. In Section 4, the energy model, phases, architecture, and algorithm of RCER are introduced. Section 5 presents the analysis of RCER. The criteria for the performance evaluation of RCER in comparison of existing schemes are discussed in Ssection 6. In the end, Ssection 7 concludes this research article.

## 2. Motivations and related work

In WSN applications [30–33], the limited resources of sensor nodes highly impact on the performance of data delivery and network stability. To amend the adjustment among data routing and energy consumption, suitable architecture is needed for the chosen data forwarders with minimum network overheads [19,34,35]. A traditional cluster-based protocol LEACH[36] has been presented, however, such solution depends on generating random clusters, which results in unbalanced energy consumption among nodes and role of cluster heads (CHs) are not evenly distributed across the network field. In addition, for the enhancement of LEACH protocol, many solutions [37–39] have been proposed. Although, such solutions improved network lifetime in the comparison of earlier schemes, however, incur further communication overheads and unbalanced energy consumption. In addition, the authors developed Partition-based (pLEACH) algorithm [40], purposes for prolonging network lifetime. Like other traditional routing protocols, pLEACH also divided into two main phases and comprises of different rounds. pLEACH firstly divides the network field into different sectors then select the highest energy level nodes as CHs based on centralized calculations. However, construction of sub-optimal panels and non-optimized route discovery lead to energy consumption in an unbalanced manner.

Different schemes [41–45] have been proposed, that generate clusters of unequal sizes. The target of such schemes is to address the problem of the energy hole. Usually, the nodes that are nearest to Base Station (BS), have to deal with high rate data receiving and forwarding, which results in reducing network throughput and lifetime. Therefore, different solutions have been developed to generate unequal sized clustering, which aims to construct smaller size clusters nearer to BS. However, the traffic load among CHs is not evenly distributed thereby results in inefficient load balancing. Moreover, the competitive CH election approach generates high messages overhead and consumes extra energy. In [10], authors proposed a ring zone based routing protocol (RARZ) for WSN, aims to improved energy consumption in the sensor field. The sensor nodes are partitioned in different rings around BS. In addition, data communication occurs in a multi-hop manner via the selection of next-hops. However, authors overlooked link quality factor in routing decision, which is also an important factor due to limited constraints of WSNs. Furthermore, the focal points of data forwarding are not updated based on network conditions.

In [46], authors presented a tree based aggregation algorithm for improving the energy efficiency of WSN. The construction and maintenance of the network tree are initiated by BS and known as the root node. During data aggregation and forwarding to BS, each source node determines its next-hop based on residual energy and hop-count parameters. However, this process takes a lot of time for data routing and increases end-to-end ratio. Furthermore, the constructed routes are not evaluated in terms of link quality that arise frequency of re-transmissions. On the other hand, an energy efficient scheme, Tree-based Clustering (TBC) [47] is presented, to structure the nodes in a tree-based manner based on distance factor. Every member node sends its sense data to the parent node until arrived at CH. This approach has improved data delivery performance within clusters but like LEACH the clusters formation process in TBC is same thereby results in uneven energy consumption. In addition, the aggregated data from CHs is forwarded to BS using single-hop, which results in a longer delay ratio. Authors in [48] presented Enhanced Threshold Sensitive Stable Election Protocol (ETSSEP) for heterogeneous network. The proposed protocol improved network lifetime as it changed the cluster head selection process based on dynamic probability function. The selection process of cluster head is exploit the factors of residual energy and number of cluster per round. Although, the routing paths are non-optimal and link evaluation is overlooked, which results in decreasing data delivery performance and network reliability.

In Energy efficient Heterogeneous Cluster scheme [49], authors improved network lifetime based on the weighted probability for CH election. Although the proposed scheme makes use of heterogeneous nodes with respect to residual energy and optimizes the energy depletion, however, the routing paths are not optimum towards end points. In Fault-Tolerant Energy-Efficient Clustering (FT-EEC) [50], each cluster further is divided into several small squares and only one node based on the highest residual energy is active in each square to sense the information. In this way, a minimum set of cluster members is elected for information gathering to avoid the probability of sensing hole. Moreover, if BS does not receive an ACK packet after the expired of the fixed timer, then it is assumed that CH is failed, this new node is elected as CH based on residual energy. Although, FT-EEC exclusively focus on improving a number of communications by identifying faulty nodes, however, lack to determine optimized route discovery and maintenance, which results in longer transmission delay and vulnerable to high energy consumption. On the other hands, the authors in Link-aware Clustering Mechanism (LCM) [51], contributes to the improvement of network lifetime. Although, the presented solution determines reliable paths by incorporate link quality factor in routing decisions. However,the due to performing re-clustering on a regular interval, the proposed solution required additional energy utilization and communication overheads.

## 3. Limitations of existing solutions and problem definition

Based on the aforementioned literature work, it is seen that efficient energy utilization with reliable routing is a major research concern. It is observed that most of the existing solutions are not able to adjust routing performance according to the dynamic environment and limited resources of WSN. Moreover, the recent work lacks the selection of next-hop based on the optimum decision and such solutions degrade network-wide routing performance. Furthermore, during data relaying, routing paths are restructured periodically, which is the additional overhead in terms of time consumption and transmission cost. This overhead exists due to the periodic exchange of routing and control messages in the network field. Moreover, in high nodes density scenario, most of the existing work produces network congestion and increases packet lose ratio. Therefore, the domain of energy efficiency focused for data collection and forwarding has to explore with a light-weight solution to improve network lifetime with the stable delivery ratio. Accordingly, to overcome aforesaid problems, the goal of this research article is to develop the energy-aware with reliable routing protocol for heterogeneous networks, which construct geographical sized clusters by using nodes location. Moreover, the proposed protocol uses up-to-date neighbor’s information to discover more energy efficient, shortened and less congested routing paths based on a fitness function. The fitness function comprises multiple criteria related to residual energy, hop-count, and the weighted value of RTT. The RTT factor performs a vital role in avoiding inaccessible and faraway neighbors in routing decision and decreases the chances of frequent route re-discoveries. Since the value of RTT changes with respect to time, thus proposed protocol computes its weighted value. Furthermore, instead of flooding route request RREQ packets in the entire network field to achieved data delivery, the proposed protocol transmits unicast message towards selected next-hop, which reduces routing overheads and additional energy consumption. Consequently, the proposed protocol provides the reliable next-hop selection, which significantly impacts the data forwarding process and improved network stability period. In addition, to growth route lifetime and data delivery performance, over burden wireless channels and nodes are identified, accordingly routing paths are re-structured based on the measurement of communication links and nodes abilities.

## 4. RCER protocol

This section presents the detail description of the proposed protocol RCER.

### 4.1. Assumptions

The typical network is presumed with certain network assumptions that are as underlined.

- All the nodes have unique IDs, deployed randomly in sensor field and remain immobile.
- By exploring GPS or position algorithm, sensor nodes are location-aware.
- Heterogeneous nodes may have more energy resource as compared to normal nodes.
- All normal nodes have the same capabilities and constraints.
- The transmission power of sensor nodes may be adjusted by employing receiver distance.
- Sink node or BS is prosperous in resources as compared to other nodes and has a long range radio transceiver.

### 4.2. Energy model of RCER protocol

This section presents the energy model for proposed RCER protocol for heterogeneity nodes in terms of their energy. Nodes are randomly organized in the sensor field and *n* is a fraction of heterogeneous nodes that have additional energy in the comparison of normal nodes. During data forwarding, only elected CHs are responsible for transmitting sensory information to BS via multi-hop path.

By exploiting radio energy consumption model[36], the required energy for *l* sized data packet of distance *d* among source and destination is shown in Eqs (1) and (2).

$$E_{Tx}=\left\{ \begin{array}{c} (E_{elect}+{d^{2}*E}_{amp1})*l,\;d\leq d_{t}\\ (E_{elect}+{d^{4}*E}_{amp2})*l,\;d˃d_{t} \end{array}\right.$$ (1)

$$E_{Rx}=E_{elect}*l$$ (2)

The consumed energy in transmitting and receiving a particular data bit is denoted by *E*_*elect*_. On the other hand, based on the distance threshold *d*_*t*_ between source and destination, a transmitter amplifier’s energy consumption is presented by *d*^2^ * *E*_*amp*1_ or *d*^4^ * *E*_*amp*1_.

### 4.3. Architecture of RCER

**Fig 1:**
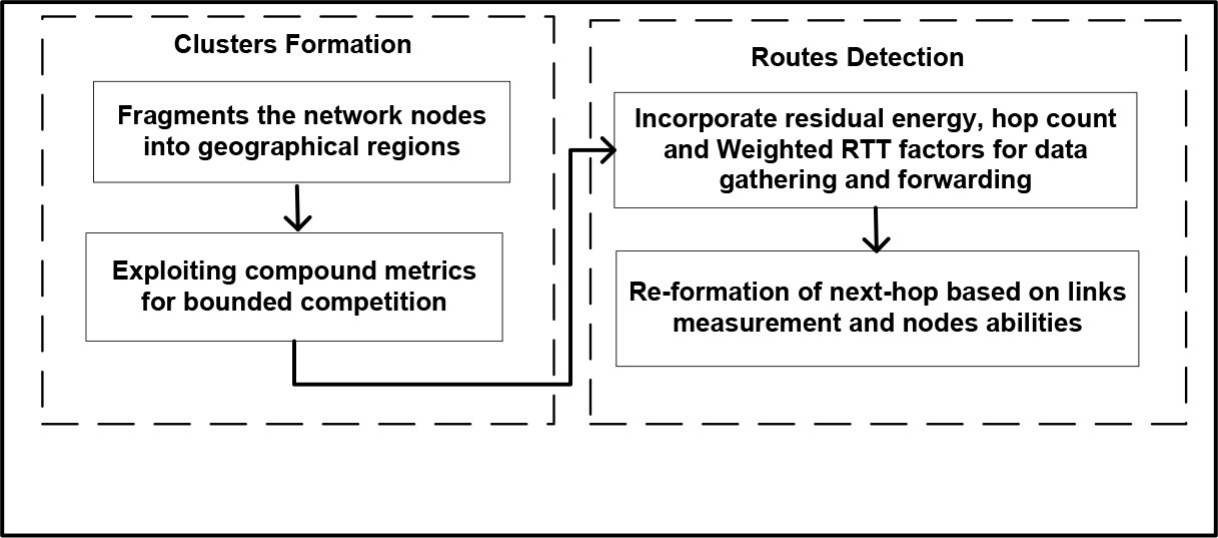
Design of RCER protocol.

The design of RCER constitutes clusters formation and routes detection phases as shown in “Fig 1”.

- Firstly, the geographical sized clusters are constructed in the network field by using nodes locality and region based election is initiated. Moreover, the consideration of heterogenous nodes in term of energy has a significant impact on network performance.
- Next, in order to indicate probable next-hops for forwarding sensory data, the constructed clusters by RCER are used by the optimized route detection phase. The optimized route detection phase is responsible for determining the shortest routes and contributes to excluding energy de-efficient nodes. Furthermore, to measure the network traffic on the wireless channels, a weighted value of RTT is also comprised in routing decision.
- In the end, the performance of wireless links and nodes status are measured and accordingly routing phase resumes on the newly prepared data trasnmissions paths. All the three main phases of RCER are collectively combined to achieve reliable routing with improved energy efficiency.

### 4.4. Phase I: Clusters formation

In most of the existing solutions, the entire sensor field is structured into discrete regions randomly, which results in imbalanced energy consumption and load distribution. To achieve energy efficiency, RCER protocol originates the construction of clusters by making use of nodes locality. Initially, BS floods its discovery message in the sensor field. Next-hop nodes store the BS discovery information and update their routing tables. Subsequent, the BS discovery information is further disseminated to neighbors in a structured manner. Like this, all the nodes update their routing tables by choosing suitable neighbor based on least hop counts. Once the construction of routing table is over, sensor nodes choose next-hop and propagate their location coordinates. The same procedure continued until BS has an inclusive statistics of the entire network field.

**Fig 2:**
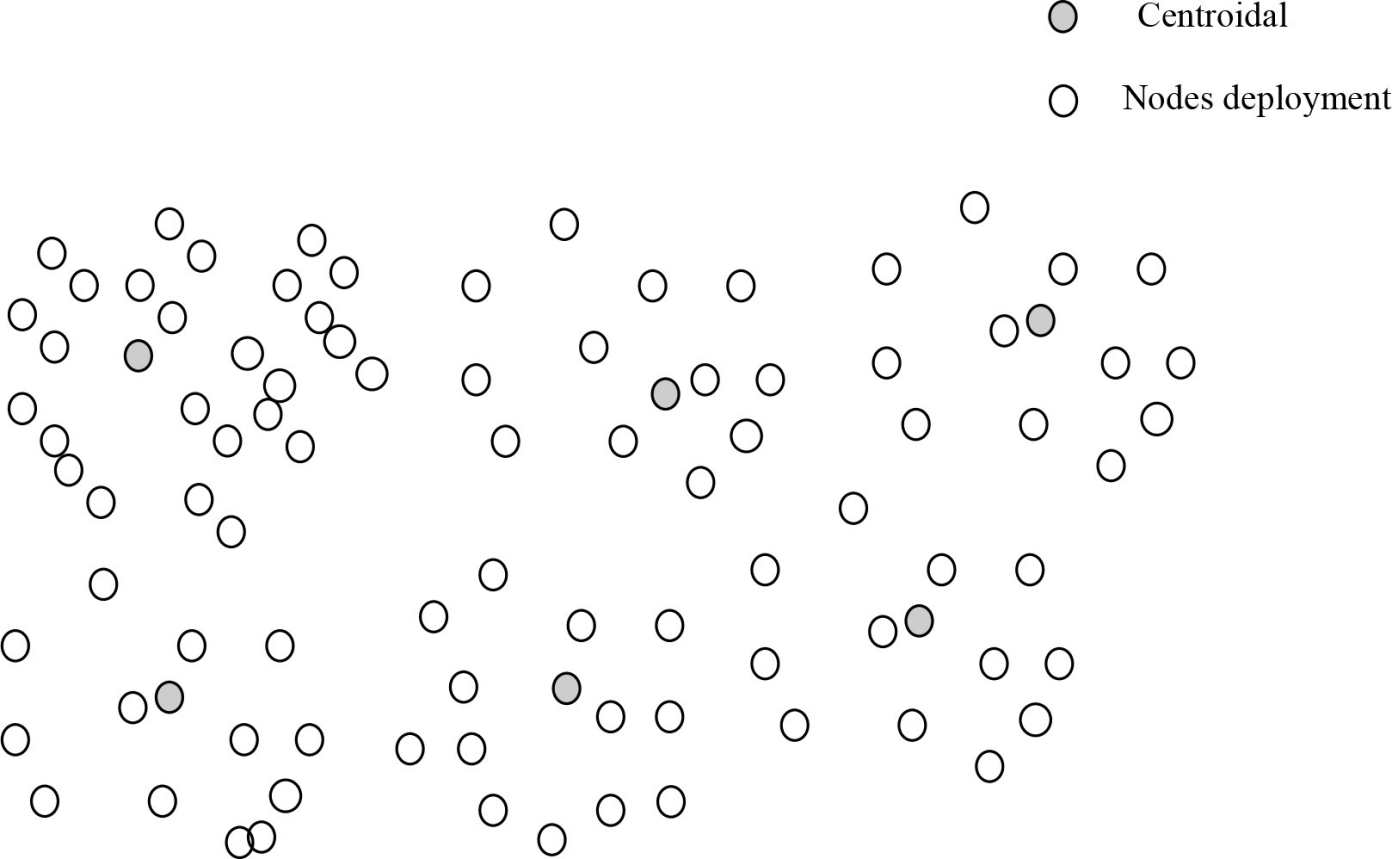
Clusters formation.

The nodes that reside in preset transmission radius *T*_*r*_, BS determines the centroidals by exploiting their locations. Next, the nodes that are closest towards centroidal are grouped into a particular cluster as shown in “Fig 2”. In this way, closest neighbors are gathered into the same group, as a result, generates energy-efficient clusters with the least clustering overheads. Subsequent, the formation of geographical sized clustering, RCER prompts the method of bounded CH election within the limit of each cluster, which decreases computational cost. The election stretagy exploits complex factors and deals with nodes centrality and residual energy facets. Furthermore, to balance the energy consumption in the network field, only those nodes are contributed in election process whose energy resource *node*_*energy*_ is exceed than an optimistic threshold *optimistic*_*threshold*_ as shown in Eq (3). Basically, the *optimistic*_*threshold*_ is adjusted dynamically based on the ratio μ of node’s energy reduction in network operations. Subsequently, among the candidates, the node centrality factor is incorporated. Basically, centrality *C*(*x*) of node *n* is the measure of its distance *d* from position *x* to its neighbors *yi* inside a particular cluster as given in Eq (4), and it is reciprocal of the sum of distance of between the node and its neighbors. The basis behind the node centrality factor is to select nodes as initial CHs that require least network overheads and energy consumption.

$$optimistic_{threshold}=\mu^{*}energy_{init}$$ (3)

C(x)=1∑i=1nd(x,yi) (4)

where *energy*_*init*_ in Eq (3) shows the initial energy of a deployment nodes.

### 4.5. Phase II: Route detection

The route detection phase comprises of two sub-components, one is the next-hop selection and other is network measurement. In the first component, the set of appropriate next-hops are identified based on multi-criteria, which provides optimum data relaying and balances the transmission load with uniform energy consumption. During routing decision, proposed RCER protocol make use of fitness function to determine Forwarder Point (FP) of node *i*, which integrates hop-count *h*_*count*_*i*_, residual energy *e*_*i*_, and Weighted Round Trip Time ${W_{RTT}}_{i,j}$ factors, as shown in Eq (5).

$$FP={w_{1}*e}_{i}+w_{2}*\frac{1}{{h\_count}_{i}}+w_{3}*\frac{1}{{W_{RTT}}_{i,j}}$$ (5)

The computed value of FP and all the three factors residual energy, hop-count, and weighted RTT are normalized in the range of (0.0,1.0). The coefficients *w*_1_,*w*_2_,*w*_3_ represent the weighting factors explicitly the residual energy, hop-count and weighted RTT. Accordingly, the node that optimizes the composite routing function in terms of energy, hop-count and weighted RTT is elected as next-hop. It might be a case that more than one next-hop have the same FP values. In such case, Node_ID breaks the ties and source node keeps an only single entry in its neighbor table. Table 1 illusrates the structure of the neighbor table. During data forwarding, the integration of energy factor in routing decision significant impacts on network lifetime with route stability. In addition, high network throughput is achieved due to the selection of least RTT next-hop. Moreover, in order to incorporate the least number of nodes during data routing, the shortest routes are formulated based on hop-counts. In order to construct a neighbor table, each source node *i* broadcasts RREQ in its transmission range and on receiving, store the residual energy and number of hops information. In addition, each node *i* determines RTT for a beacon messages *X*_*i*_ at node *j* as shown in Eq (6).

**Table 1:** Neighbor table.

Residual energy *e*_*j*_	*h*_*count*_*i*_	${W_{RTT}}_{i,j}$	*next_hop* = ${w_{1}*e}_{i}+w_{2}*\frac{1}{{h\_count}_{i}}+w_{3}*\frac{1}{{W_{RTT}}_{i}}$
--------	----------	---------	---------

$${RTT}_{i,j}=\sum \left(R_{x}-T_{x}\right)$$ (6)

RTT is used to measure the length of time that it proceeds to send data packets and the length of time that it proceeds for an ACK for data packets to be received[52]. Basically, the distance and transmission media also impact the value of RTT. In Eq 6, *T*_*x*_ and *R*_*x*_ are transmitting and receiving time of beacon message *x*. Thus, minimum *RTT*_*i*,*j*_ indicates the less congested wireless channel, which results in an improved network throughout with minimum data interruption. Moreover, both *T*_*x*_ and *R*_*x*_ are synchronized with respect to time based on the timing-sync protocol for sensor networks[53]. Time synchronization permits the nodes to snooze for a particular interval and then awaken periodically to receive beacon message. As the value of RTT diverges over time, so its weighted value ${W_{RTT}}_{i,j}$ is computed based on Eq (7).

$${W_{RTT}}_{i,j}=\alpha *{RTT}_{i,j}(t)+{\beta *RTT}_{i,j}(t+1)$$ (7)

where *α* and β are normalized factors with the ranges between 0 to 1, and t presents the integer time interval. Both *α* and β must be equal to 100% for their equal percentages. After the calculation of ${W_{RTT}}_{i,j}$, each node handovers the calculated ${W_{RTT}}_{i,j}$ to its neighbors, and consequently determined ${W_{RTT}}_{i,j}$ value is noted in the neighbor table. Subsequently, in order to choose next-hop, each source node determines FP of its neighbors and based on the result, the source node transmits a unicast message towards selected next-hop. Accordingly, uninterrupted, less congested and most energy-aware routing paths are constructed towards particular CH in each cluster. In addition, CHs are responsible for local data collection and known as a crucial end. Our RCER protocol constitutes a Backbone Formation (BF), which is responsible for electing a set of CHs for the construction of adaptive paths towards BS. The basis behind the construction of BF is to reduce the communication power of chosen CHs for data routing and source to a fair distribution of energy consumption. In addition, when particular CH drops its energy resource to *optimistic*_*threshold*_ OR upon completion of the predefined time epoch (*Δt*), the RCER protocol initiates re-election process as being exploited in aforementioned clusters formation phase.

In data routing, the inherent characteristics of low powered communication links and tightly limited energy resources of sensor nodes lead to various vulnerabilities such as unnecessary energy consumption, re-transmissions and frequently routes discoveries. Thus, the main aim of network measurement component is to enable source node for constructing an alternative route towards the destination, if the active route is no longer accessible due to encountering of any unreliable node or fragmented link. To identify unreliable nodes or fragmented links on active route, source node *i* determines the latency epoch *l* among its next-hop *j* by disseminating *n* beacon packets periodically as shown in Eq (8).

li,j=Tr−TsPn (8)

In Eq (8), *T*_*r*_ indicates receiving time of beacon message, *T*_*s*_ shows sending time of beacon message and *P*_*n*_ is a total number of beacon messages. Afterwards, a set *n* of the computed latency epoch *l*_*i*,*j*_ are summed up to fixed a latency threshold *latency*_*threshold*_ as shown in Eq (9). If the latency ratio of a particular link among two consecutive nodes is higher than the set threshold, will be considered as over congested and inappropriate for further data forwarding. Therefore, the over congested node sends a route-alter message towards source node via downstream next-hop. Source node upon receiving the route_alter message, marks the routing path invalid and removes the entry from routing table. By exploiting the aforementioned optimized routing phase, the source node selects a fresh next-hop, and unicasts RREQ packet towards the newly selected next-hop for re-continuing data forwarding on an alternative route.

$${latency}_{threshold}=\frac{1}{n}\sum_{i=1}^{n}l_{i,j(i)}$$ (9)

**Fig 3:**
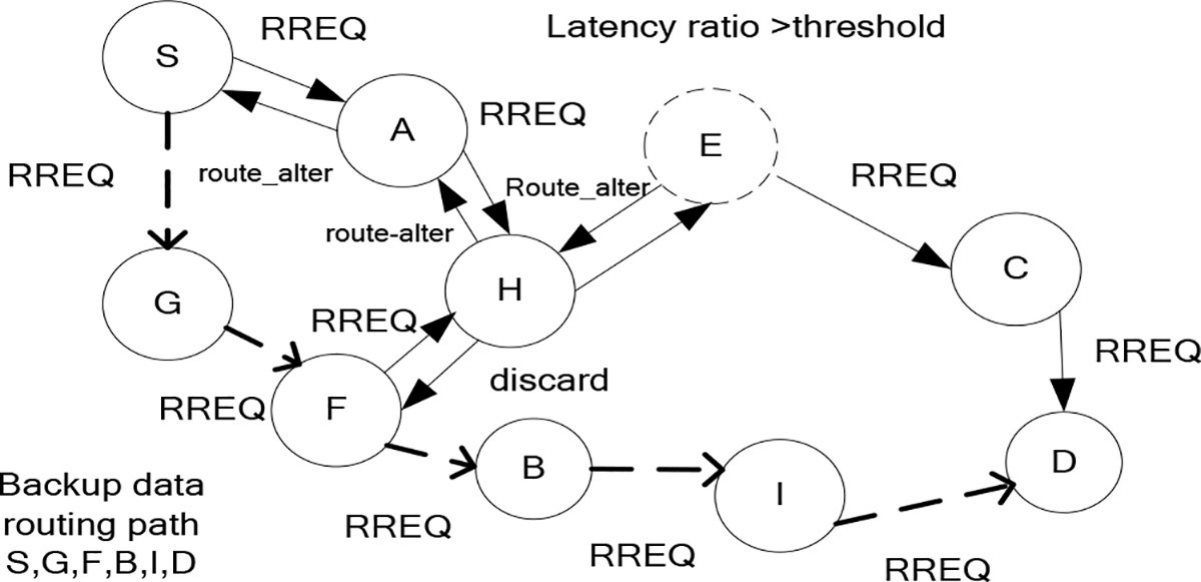
Re-formation of the backup routing path.

**Fig 4:**
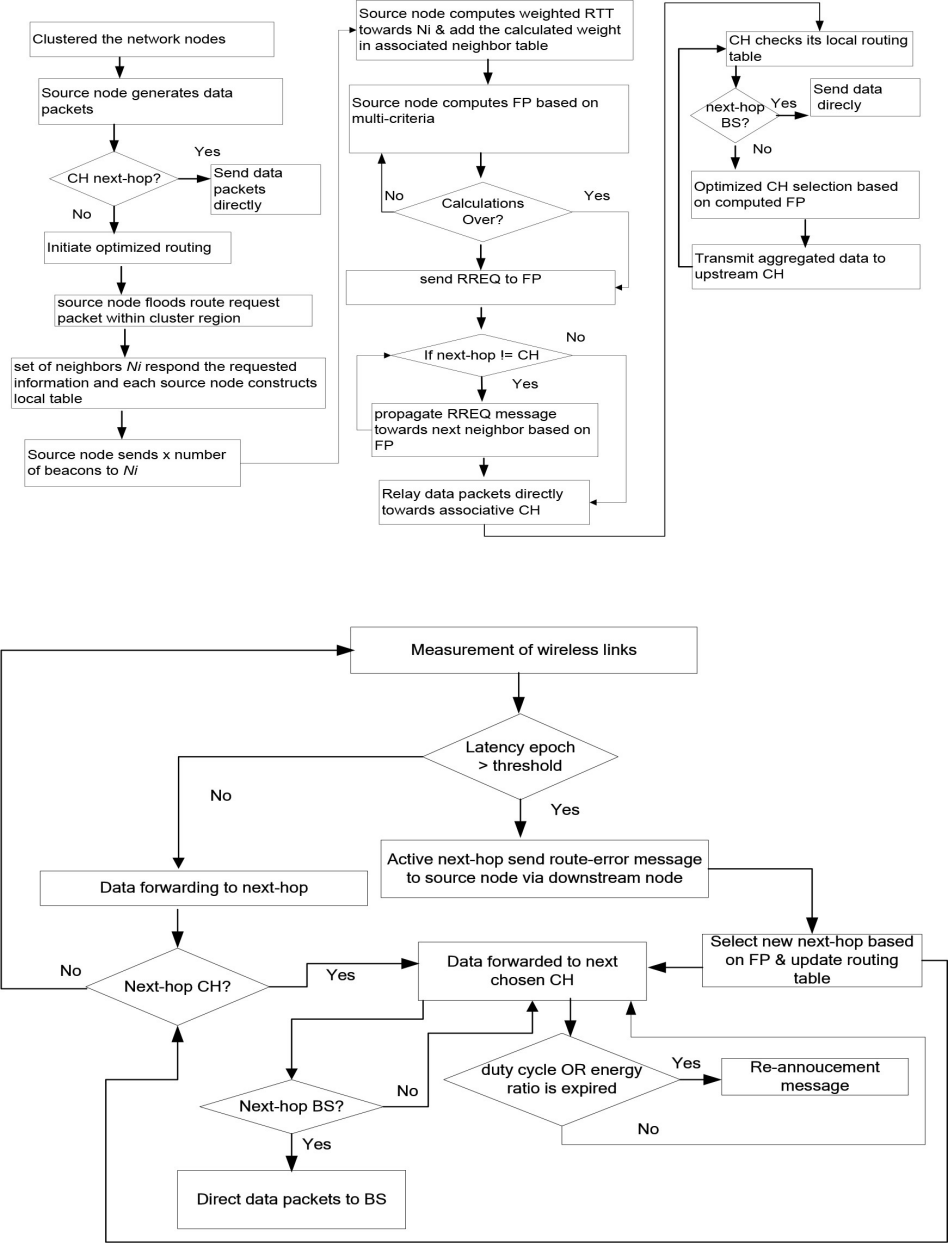
**(a)(b)**Workflow of RCER protocol.

In “Fig 3”, the latency ratio on node E is greater than certain threshold, so node E sends route_alter message towards source node S via node H and A. Afterwards, source node S re-formulate an alternative route towards destination D via node G, F, B and I. Upon receiving the RREQ packet, the chosen next-hop replied to source node with ACK message. In case, if the source node does not receive any ACK message, then the same aforementioned optimized routing phase is continued for the selection process of next-hop. In addition, to achieve uniform load distribution, each node may a part of only one active route. In case, it might happen that the node received multiple RREQ packets, in such case the duplicate packet is discarded. In “Fig 3”, node H received duplicate RREQ packet from node F, so receiving RREQ packet is discarded by node H. “Fig 4(A)” and “Fig 4(B)” shows the workflow of all the phases of proposed RCER protocol.

### 4.6. Algorithm description

Algorithm 1 governs the phases of proposed RCER protocol.

**Algorithm 1:** clusters formation, next-hop selection, and network measurement phases

1. Begin

2. **procedure** CLUSTERS

3. Determine the set of centroidals based on nodes location

4.     **for (**i = 1; i< = Nodes; i++)

5.         **do**

6.           **If *node***_***energy***_**>***optimistic*_*threshold*_

7.             **set** list_of_candidates []

8.           **endif**

9.        **end for**

10.       **for (**i = 1; i< = list_of_candidates;i++)

11.          compute the node centrality of node _i_

12.          set the max(closness node _i_) as CH _i_

13.          **end for**

14. **end procedure**

15. **procedure** route detection

16.   **for each *node***_***i***_ ∈ [1:*M*]

17.     **do**

18.         call optimized_data_routing ()

19.       **while** (y! = destination)

20.         *node*_*i*_ sets next-hop by using highest *FP*_*i*_

21.         *FP*_*i*_ Reply to *y*

22.         *y* = *FP*_*i*_

23.           **if *y***_***i***_.latency epoch is high

24.             call route_restore ()

25.           **end if**

26.       **end while**

27.     **end for**

28.   **end procedure**

29. **Procedure** nodes_status

30.           **if** (*CH*_*i*_. energy <μ*energy_*init*_)

31.               initiate re-election ()

32.           **if** (Δt expired) then

33.               initiate re-election ()

34.             **end if**

35.           **end if**

36. **end procedure**

37. **End**

## 5. RCER analysis

The following are the characteristics of the proposed RCER protocol as compared to existing schemes.

- The RCER protocol is designed for hetregeneous WSNs based on the cluster-based solution, and effective for energy efficiency with reliable routing.
- The clusters formation are achieved based on nodes region, which results in the generation of more stable clusters with minimal energy consumption.
- To achieve optimal routing decision, fitness function based on multiple factors is used. It balancing energy consumption and reduces routing overheads, as only neighboring nodes participate in routes construction procedure.
- Unlike periodic next-hops re-formation in entire network field, proposed RCER protocol updates their locations by using network measurements.
- RCER reduces the clustering overheads, as cluster setup phase executes only precise time at the start of network initialization, Afterwards, the position of CH rotates within region of each cluster. Due to limited computational processing, RCER protocol minimizes overheads and leads to improved network lifetime.

## 6. RCER performance evaluation

The section evaluates the routing performance of RCER protocol in well-known simulator tool NS2 with the comparison of existing work. During the evaluation, different experiments are performed based on a high-density node and varying network load. The performance of RCER protocol is evaluated in terms of network lifetime, energy consumption, network throughput average end-to-end delay, route lifetime and packet delivery ratio. Sensor nodes are static and randomly deployed in square size network field. The initial energy levels are assigned to nodes in the range of 2j to 5j. Simulation time is set to 1500sec to measure the performance of the proposed protocol against existing work. The transmission range of the nodes is fixed to 25m. Table 2 summarizes the default simulation parameters.

**Table 2:** Simulation parameters.

Parameter	Value
Sensor field	300 X 300m^2^
Initial energy level	2j to 5j
E_elect_	60nJ/ bit
E_amp_	20nJ/bit/m^2^
E_fs_	0.0013pJ/bit/m^4^
Packet size, k	127 bits
Channel bandwidth	10mbps
Transport protocol	UDP
MAC protocol	IEEE 802.15.4
Simulation time	1500 sec
μ	0.5
Evaluated protocols	RCER, LCM, Partition based LEACH, TBC
*Δt*	20 sec
Node’s transmission range	25m
*w*_1_,*w*_2_,*w*_3_	0.33,0.33,0.33

### 6.1. Sensitivity analysis of weighted factors

**Fig 5:**
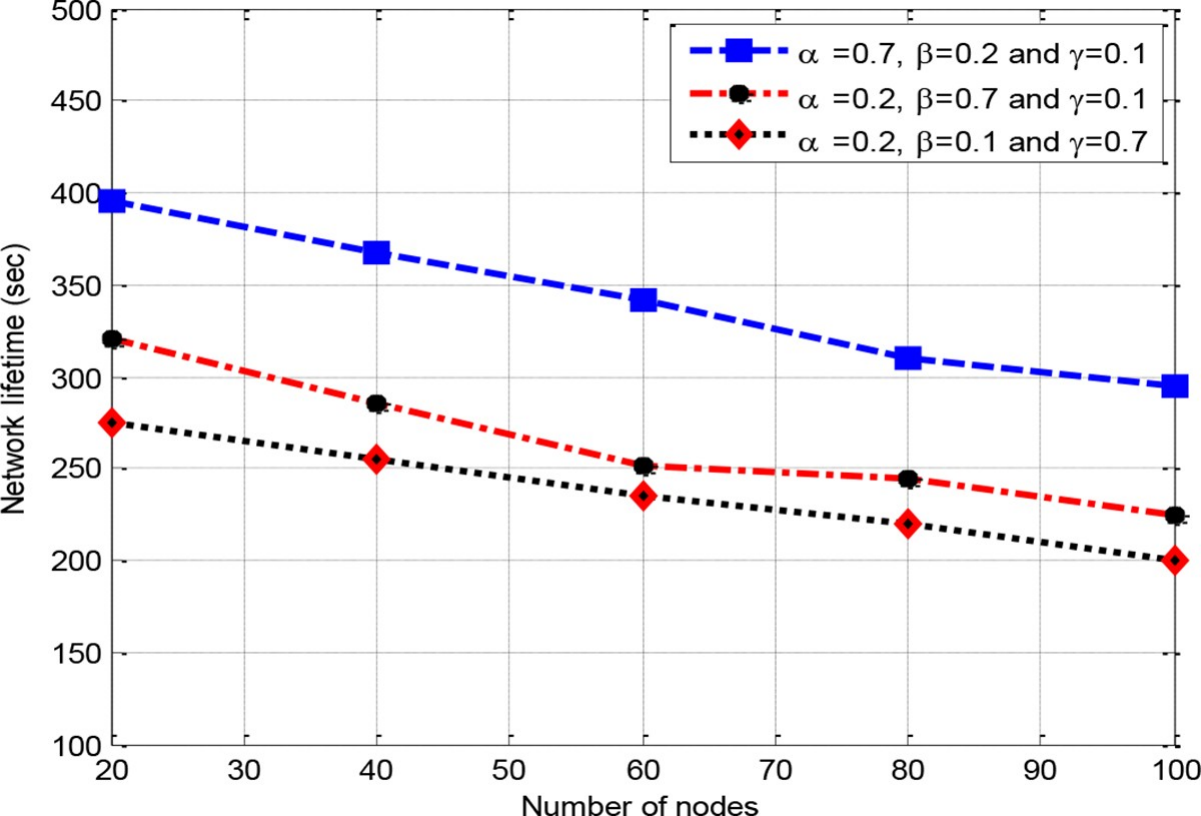
The impact of a number of nodes on network lifetime.

**Fig 6:**
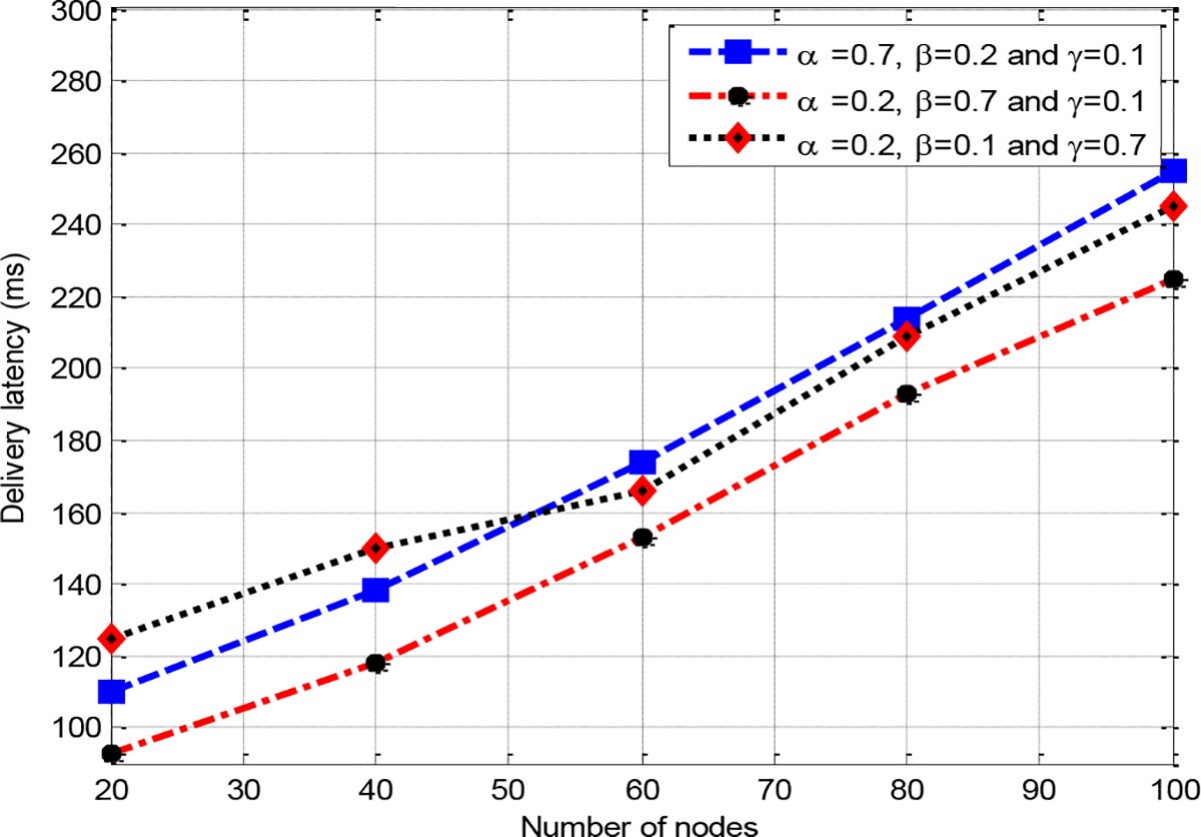
The impact of a number of nodes on delay ratio.

**Fig 7:**
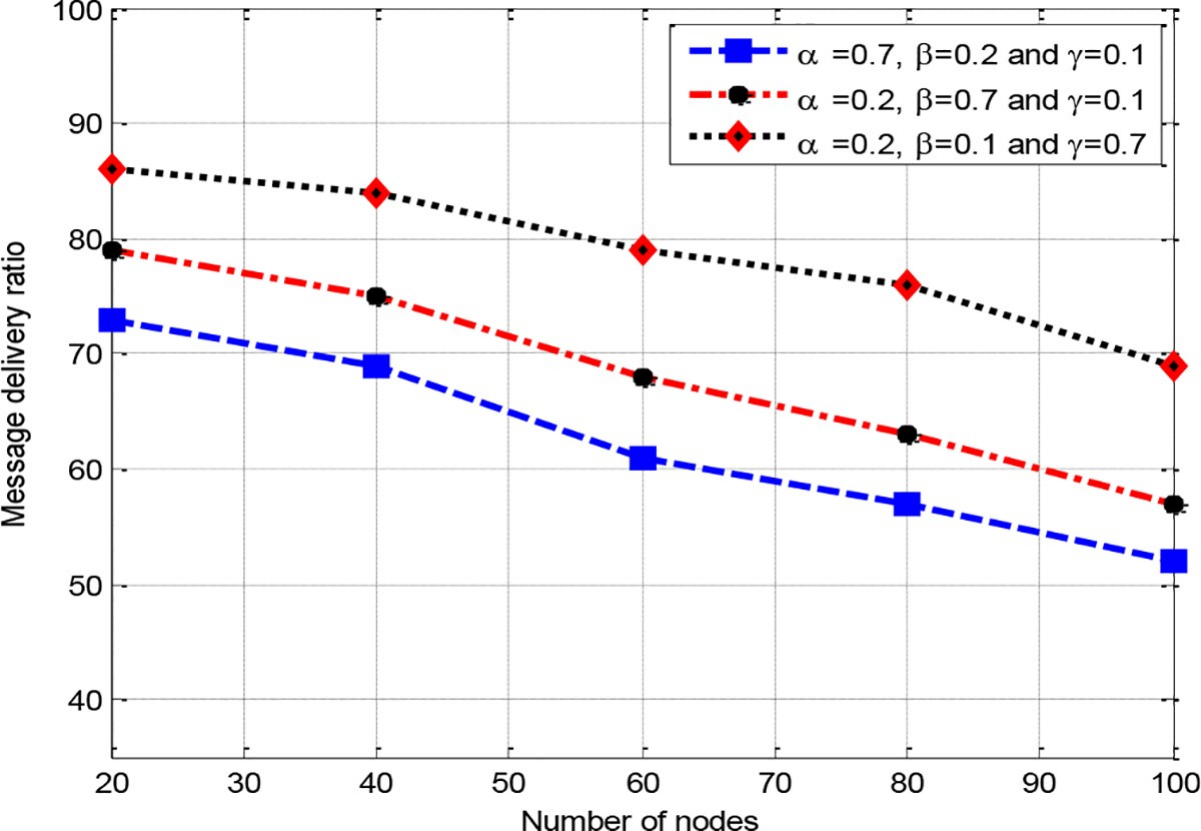
The impact of a number of nodes on network throughput.

In this section, we performed the sensitivity analysis to determine the balanced contribution between energy, hop-counts, and RTT. The weights *w*_1_,*w*_2_,*w*_3_ are assigned to energy, hop-count and RTT metrics respectively such that *w*_1_+*w*_2_+*w*_3_ = 1. Basically, the weighting values are used to highlight the percentage of each factor for makeing routing decision. Accordingly, the summation of all the percentages of weighting factors must be equal to 100%. For instance, suppose the values of *w*_1_ = 0.5, *w*_2_ = 0.3 and *w*_3_ = 0.2, indicates each weighted factor contributed by 50%, 30% and 20% respectively in the calculation of routing decision. As, to acheive optimum ratios for weighting factors *w*_1_, *w*_2_ *w*_3_, a sensitivity analysis is conducted. Therefore, a scenario is developed with three different configurations, where RCER-1(0.7,0.2,0.1) corresponds to configuraion of *w*_1_ = 0.7, *w*_2_ = 0.2 and *w*_3_ = 0.1, RCER-2 (0.2,0.7,0.1) denotes to configuration of *w*_1_ = 0.2, *w*_2_ = 0.7 and *w*_3_ = 0.1, and RCER-3(0.2,0.1,0.7) represents the configuration of *w*_1_ = 0.2, *w*_2_ = 0.1 and *w*_3_ = 0.7. In "Figs 5–7“, three assumed configurations RCER-1(0.7,0.2,0.1), RCER-2 (0.2,0.7,0.1) and RCER-3(0.2,0.1,0.7) are measured. All the three configurations are evaluated with respect to network lifetime, delay ratio and network throughput scanarios. “Fig 5” shows that RCER-1 achieves better results with respect to network lifetime where high value is given to factor energy *w*_1_. The result of delivery latency is depicted in “Fig 6”, accordingly, configuration RCER-2 results in bettter performance in comparison of RCER-1 and RCER-3, this is because of giving high weightage to hop-count parameter *w*_2_. In “Fig 7”, the configuration of RCER-3, intermediate nodes are selected based on lower RTT factor *w*_3_.

Based on the evaluation of “Figs 5–7”, it is obvious that each weighting factor has its negative and positive impact on the performance of RCER’s route discovery. Therefore, if all the weighting factors are assigned even representation, a more balanced contribution is achieved towards the optimized routing process (*w*_1_ = *w*_2_ = *w*_3_ = 0.333, however *w*_1_+*w*_2_+*w*_3_ = 1). As a result, energy, hop-count and RTT metrics are given equal impact which results in construction of shortest, energy efficient and less congested routing paths. In all following simulation experiments, RCER assigns identical values to *w*_1_, *w*_2_ and *w*_3_.

### 6.2. Simulation results

In following sub-sections, the performance of RCER protocol is evaluated in the comparison of ETSSEP, Partition-based LEACH, TBC and LCM schemes.

#### 6.2.1. Network lifetime analysis

**Fig 8:**
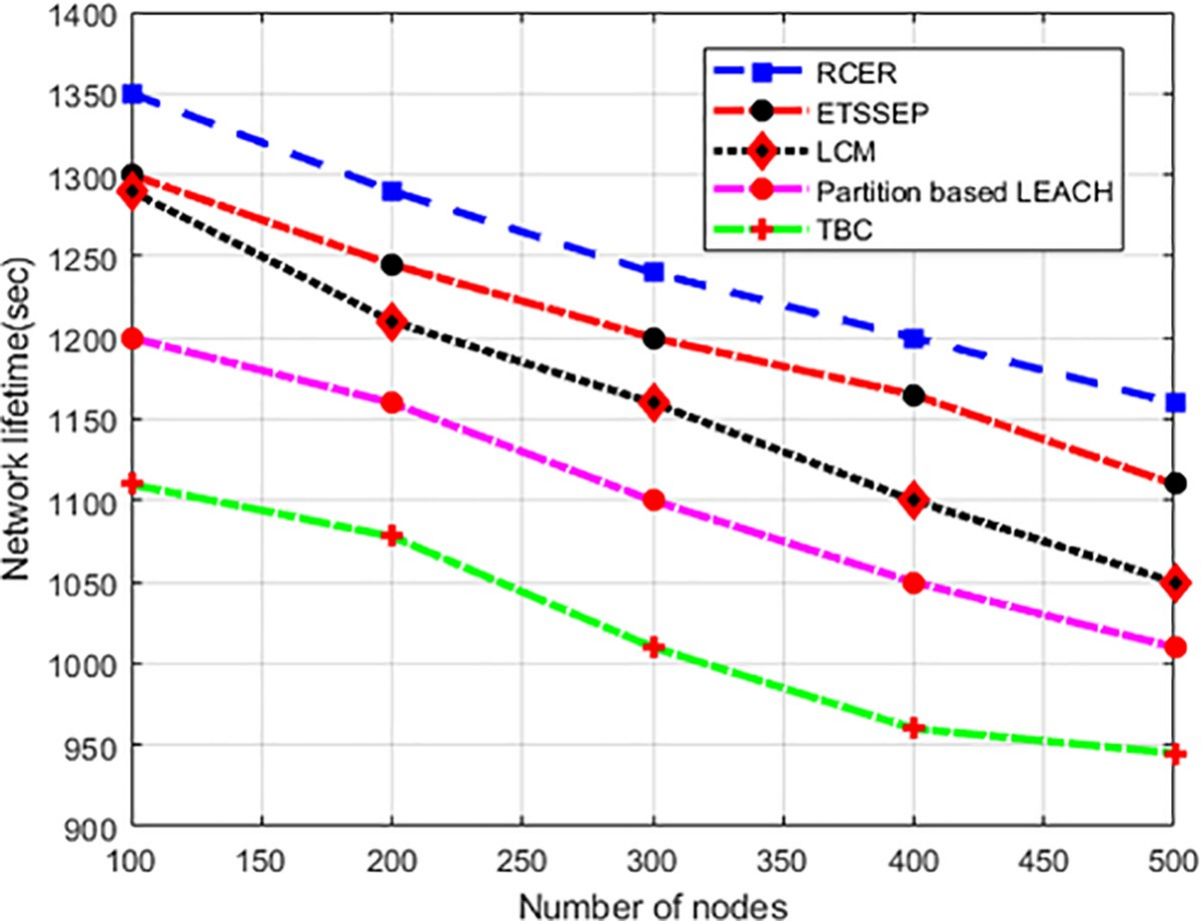
Network lifetime in high-density nodes.

For a varying number of nodes scenario, “Fig 8” evaluates the network lifetime of RCER protocol in comparison with existing schemes. It is obvious that RCER protocol has superior performance, like 11.5%, 12.2%, 14.7%, and 23% improvement is achieved in network lifetime. Unlike ETSSEP, TBC, LCM and Partition based LEACH, RCER protocol constructs geographical sized clusters and initiates CHs election mechanism among a narrow nodes. Moreover, the most energy efficient, shortest and less congested next-hops are selected to pursue data forwarding. Furthermore, the overloaded and faraway next-hops are avoided during data forwarding, as a result, RCER protocol improves network lifetime remarkably.

**Fig 9:**
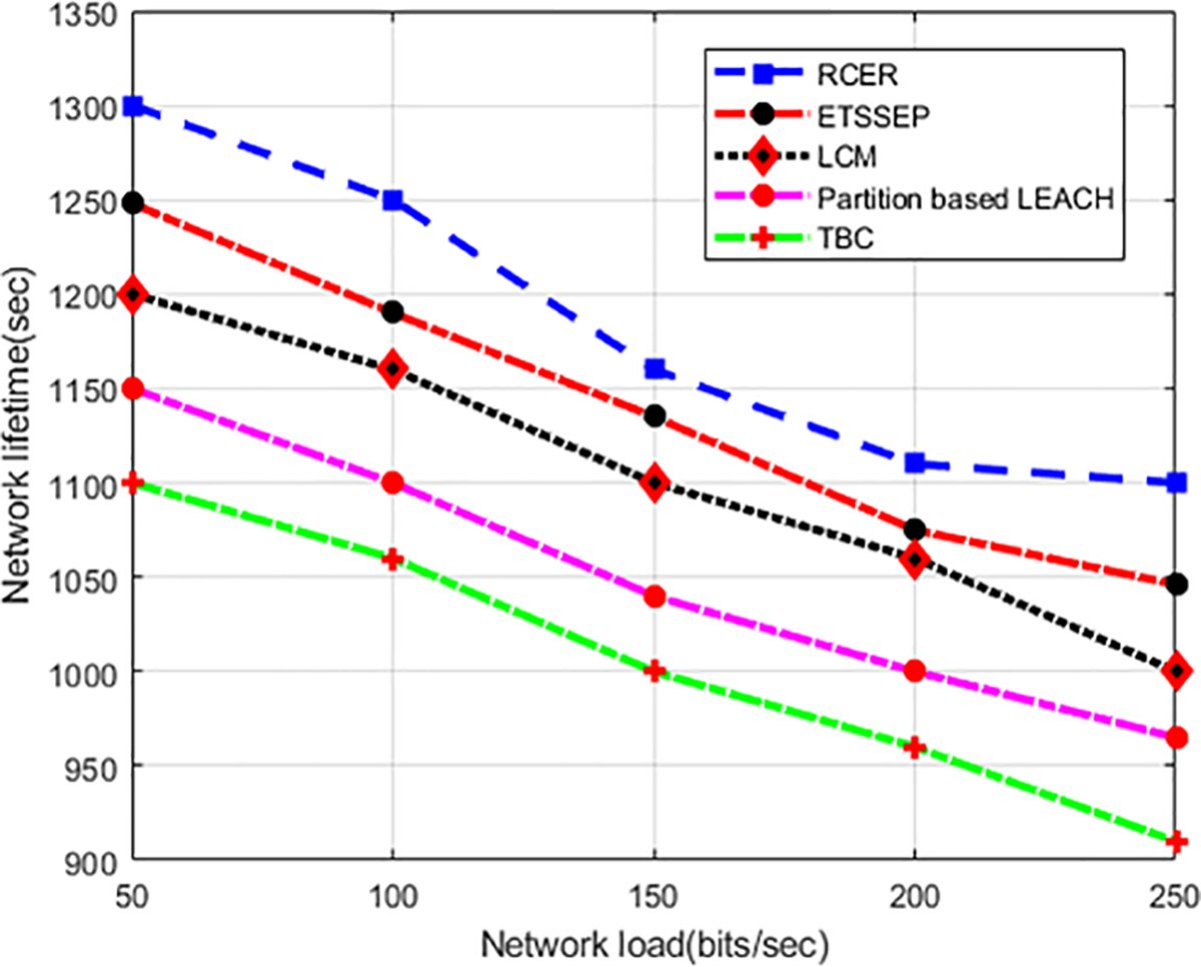
Network lifetime in varying network load.

For varying network load scenario, “Fig 9” measures the network lifetime of RCER with other existing schemes. Observably, the data traffic increases by employing more network load. On the other hand, more network load also decrease network lifetime. RCER protocol improved network lifetime by 9.6%, 10%, 20% and 39% than present solutions. This is due to considering of position factor in the generation of clusters, and the position of CHs is shifted based on demand rather than periodically, thus RCER significantly reduces the rate of energy consumption and extending network lifetime.

#### 6.2.2. Energy consumption analysis

**Fig 10:**
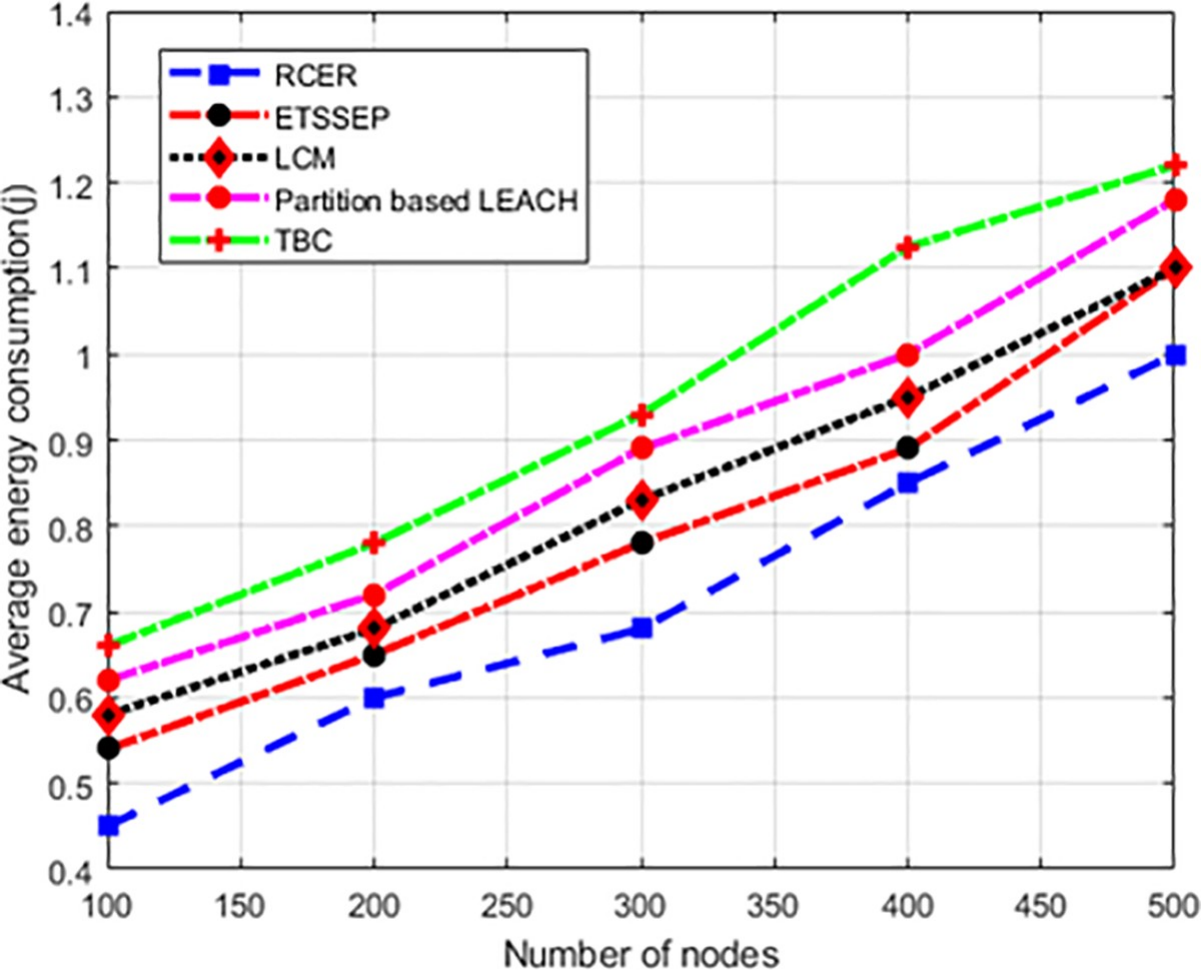
Energy consumption in high-density nodes.

“Fig 10” depicts the behavior of RCER protocol with the comparison of existing schemes for average energy consumption scenario in terms of a different number of nodes. Based on results, it is seen that RCER protocol broadly shortens the network energy consumption by 14%, 15%, 33%, and 46% respectively than existing schemes. Unlike ETSSEP, TBC, LCM and Partition based LEACH, RCER protocol reduces the computational and communication overheads, as CHs election mechanism occurs within the constrained regions. Furthermore, few numbers of nodes are accountable for in routing decision thus achieves balanced energy consumption.

**Fig 11:**
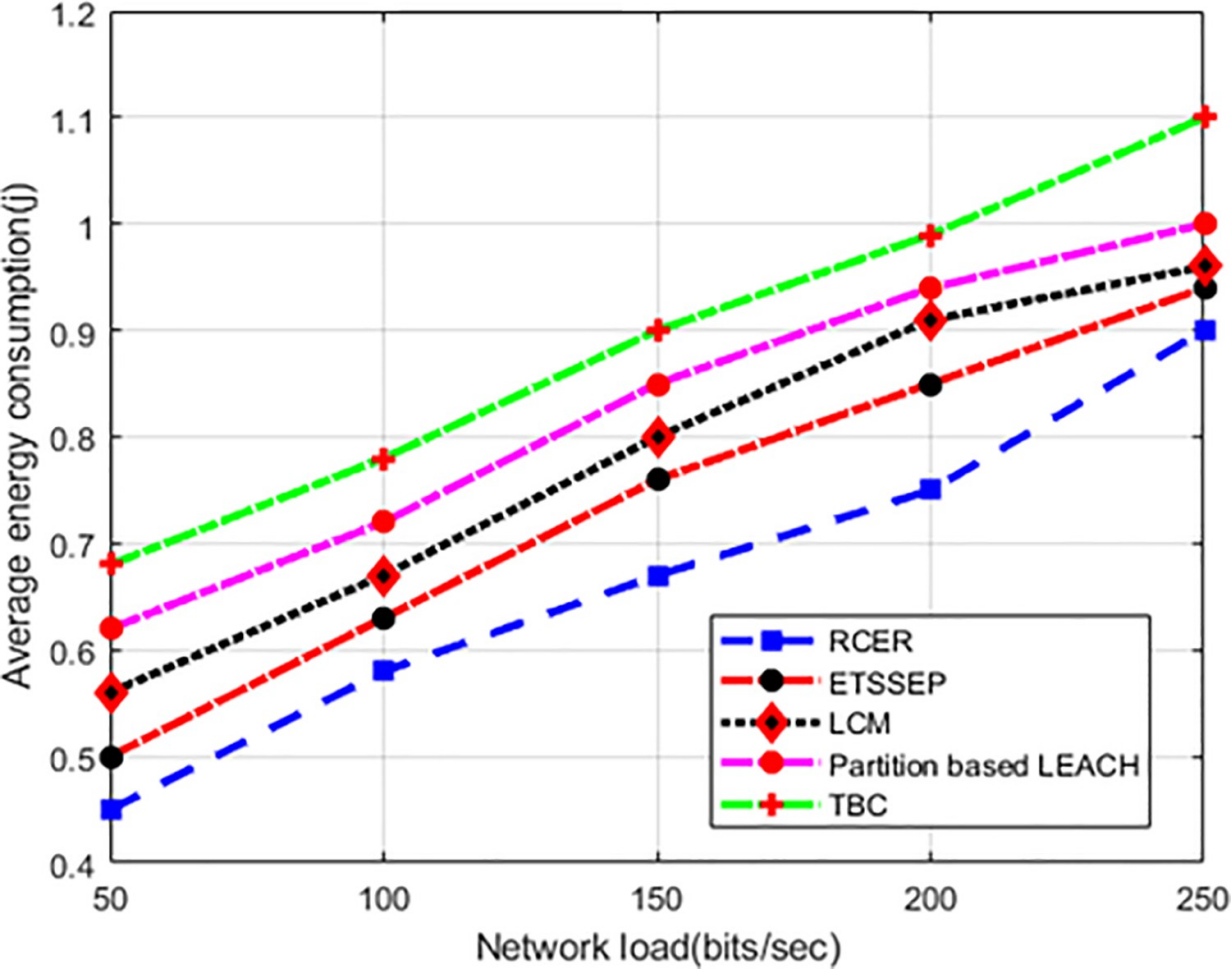
Energy consumption in varying network load.

“Fig 11” depicts the improved performance of energy consumption by 24%, 30%, 47%, and 52% respectively in terms of varying network load. The logic behind consuming lessen energy consumption is due to balance the network load among next-hops. In addition, only a restricted number of nodes come to the election process and changed their positions based on network measurement. Furthermore, incorporating the link delay factor in routing decision highly reduces the number of re-transmission, which result in remarkably decrease energy consumption.

#### 6.2.3. Network throughput analysis

**Fig 12:**
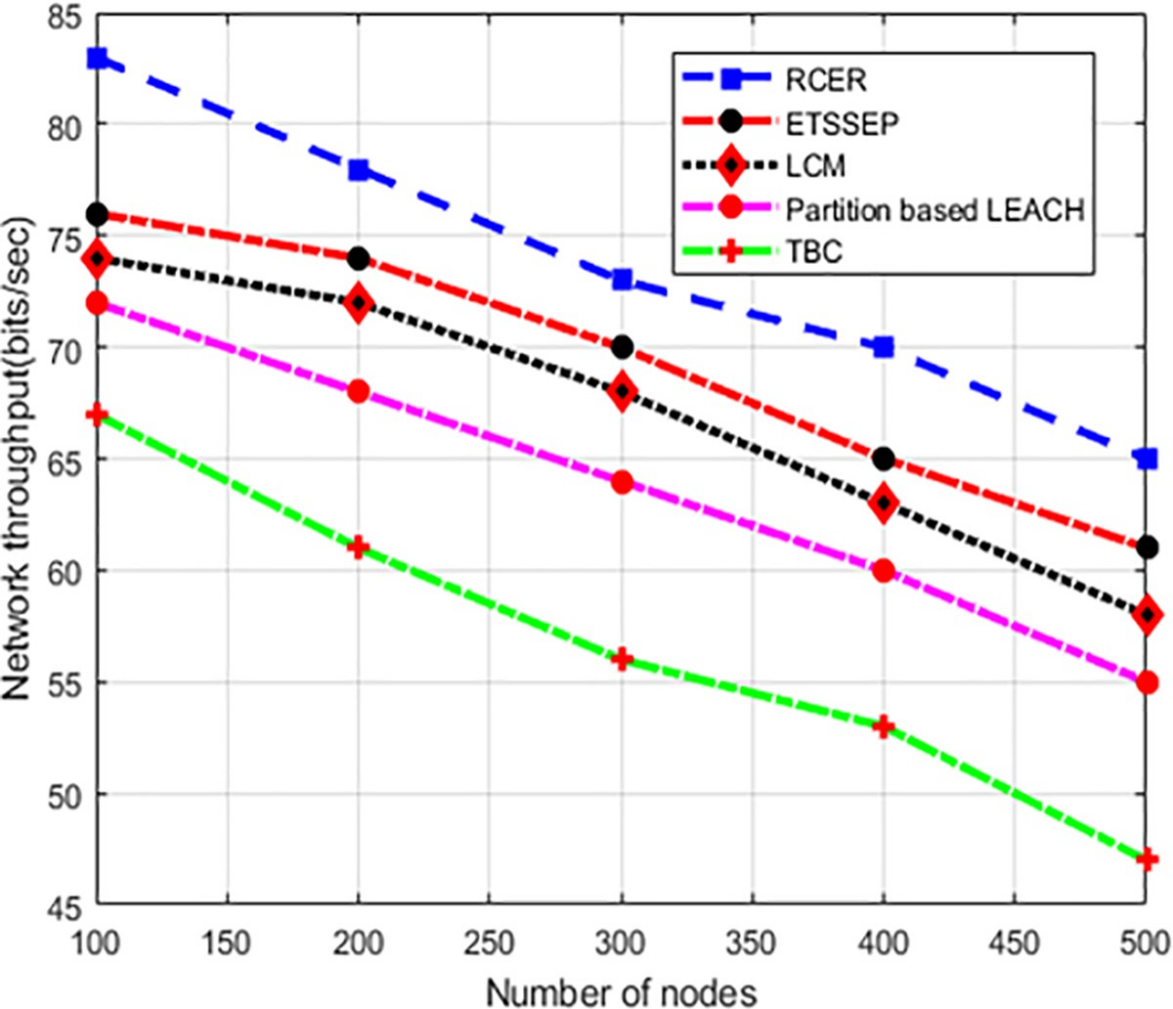
Network throughput in high-density nodes.

In “Fig 12”, the performance of RCER against existing work is compared in terms of network throughput, while considering a different number of nodes. The simulation results show that RCER achieved higher network throughput by 17.5%, 19%, 27%, and 36% with a comparison of existing schemes. This is due to that ETSSEP, TBC, LCM and Partition based LEACH perform non-optimum routing decision for the selection of next-hop, whereas RCER protocol selects next-hops by considering multi-criteria. As a result, the less congested, reliable and energy efficient nodes are chosen for the forwarding of data packets. Thus, more significance is given to neighbors which have the lowest number of hop counts, highest residual energy, and lowest RTT. The routing decision of RCER protocol not only lessens the length of the communication path and saves energy resource but on the other hand, also construct a reliable path thus improving network throughput.

**Fig 13:**
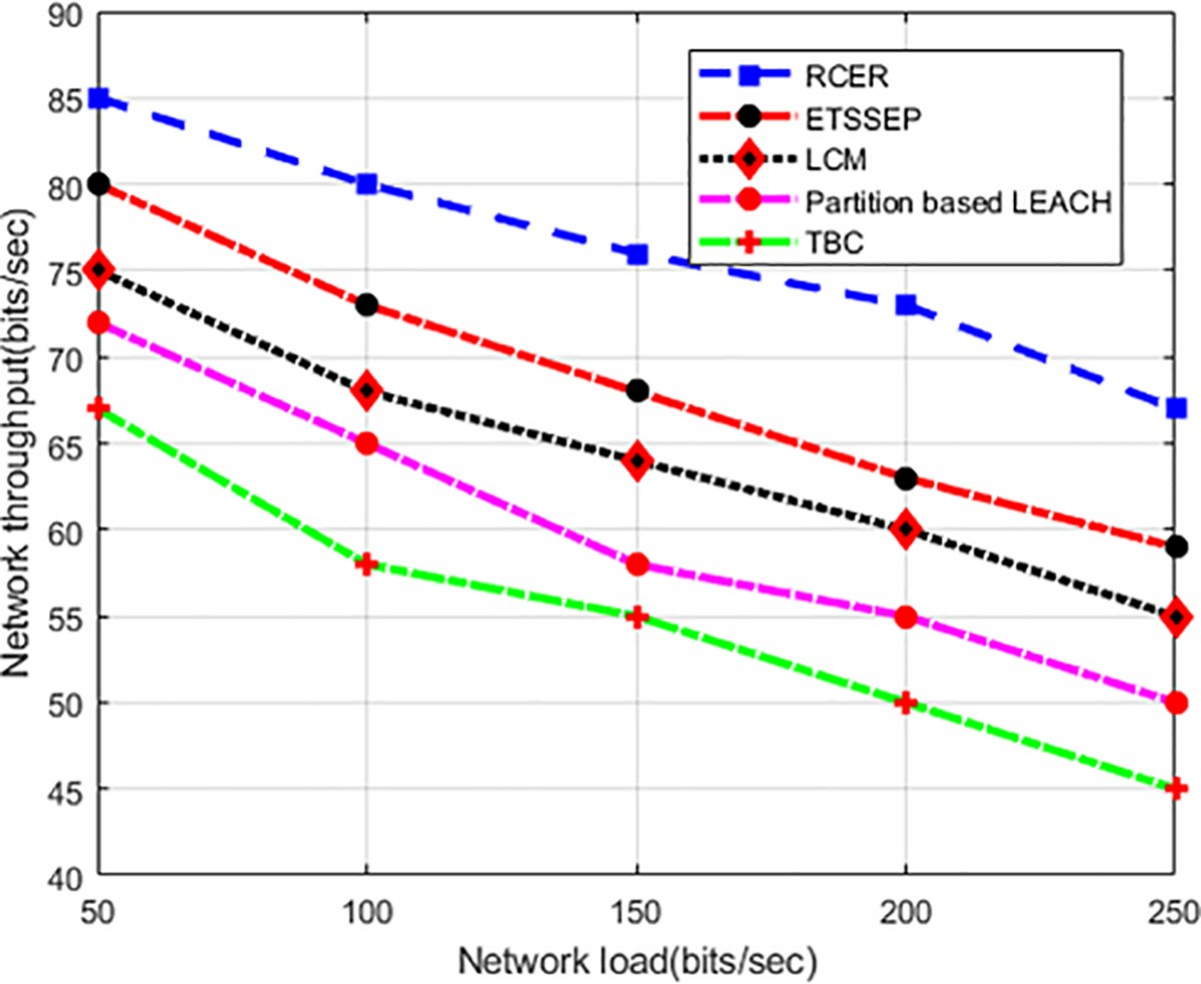
Network throughput in varying network load.

In varying network load scenario, “Fig 13” illustrates the performance of RCER with respect to network throughput in the comparison of other solutions. In fact, a higher network load in elevated traffic burden reduces network throughput and routing performance. Based on results, it is seen that RCER protocol has the highest data delivery performance, like 17%, 18%, 29%, and 37% improvement is attained than existing work. This happens because of the selection of multi-criterion data forwarders, and position of next-hop is re-formulated based on network measurement. Moreover, by considering link delay factor in routing decision, decreases the network congestion with a numeral of re-forwarding that have a significant outcome on data delivery performance.

#### 6.2.4. End-to-end delay analysis

**Fig 14:**
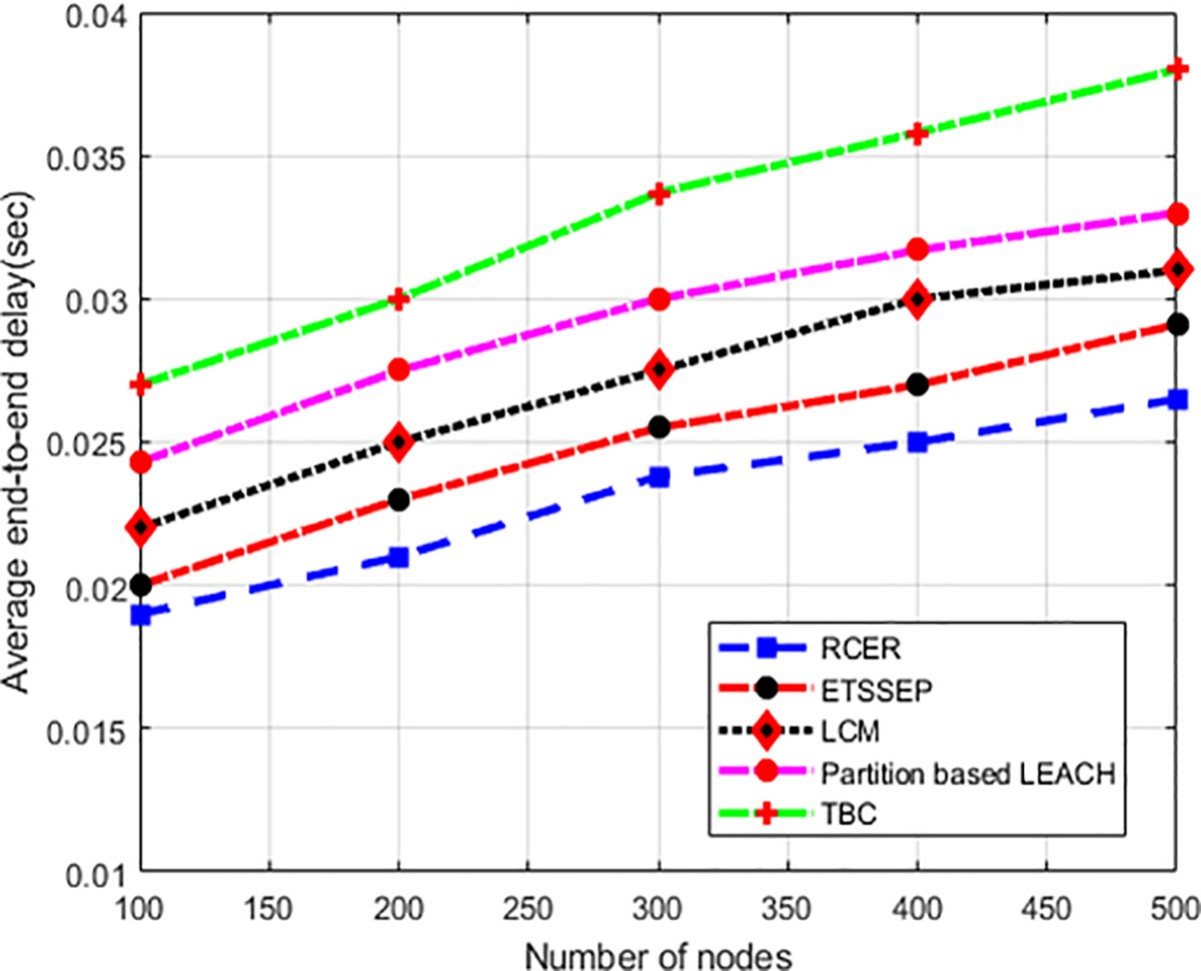
End-to-end delay in high-density nodes.

The performance of RCER with a comparison of existing work in terms of their end-to-end delay is illustrated in “Fig 14”, by considering a varying number of nodes. The simulation results show that RCER achieved 9%, 10%, 16%, and 23% reduction in average end-to-end delay. Unlike ETSSEP, TBC, LCM and Partition based LEACH, RCER routing conclusion is more adaptable in the dynamic scenarios and reduces the probability of route breakages and re-data-forwarding. Moreover, by computing links performance based on latency epoch, overloaded communication paths are identified and consequently, the next-hops are re-adjusted. Accordingly, the partial re-formation of next-hop instead of constructing the entire routing path significantly minimizes time consumption and data delivery interruption.

**Fig 15:**
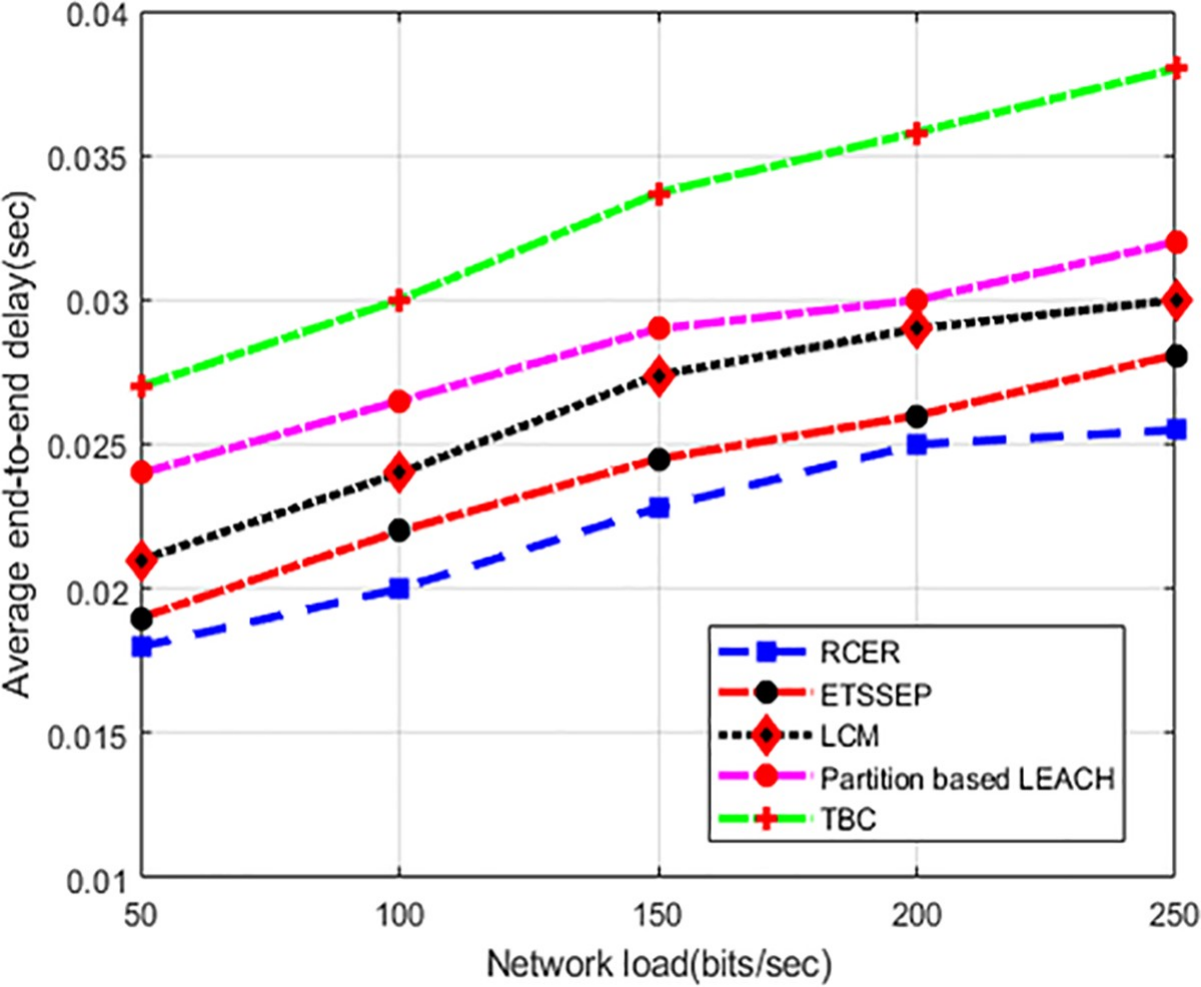
End-to-end delay in varying network load.

Average end-to-end delay in varying network load scenario is depicted in "Fig 15”. RCER protocol improves the performance of network delay by 18%, 19%, 32%, and 40% in the comparison to existing work. This happens because the construction of the route in RCER is more active in terms of multi-facet attributes. Moreover, in routing decision, only that particular next-hops are chosen, those condense the delay ratio with minimum network overheads and time consumption. In addition, RCER avoids to choose faraway neighbors and dropping the number of trails while sending data packets.

#### 6.2.5. Route lifetime analysis

**Fig 16:**
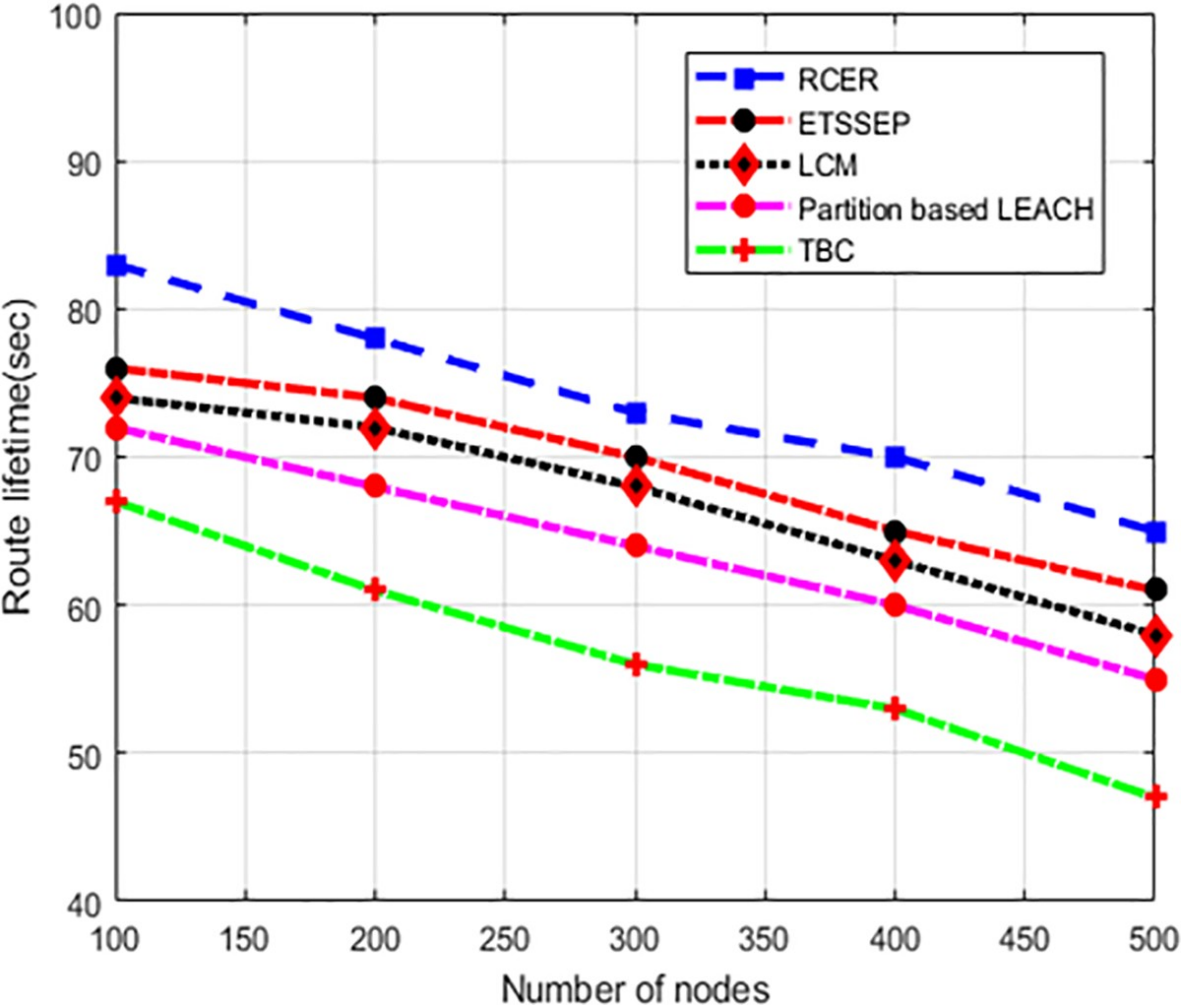
Route lifetime in high-density nodes.

In a varying number of nodes scenario, “Fig 16” illustrates the route lifetime in comparison of existing schemes. As can be observed from the simulation results that RCER achieves longer route lifetime than existing schemes by 15%. 16%, 23%, and 34%. The reason behind such performance improvement of RCER is due to the incorporation of nodes abilities and link delay factor for routes re-amendment, whereas ETSSEP, TBC, LCM and Partition based LEACH schemes re-structured the routing paths periodically without considering network conditions. Moreover, the nodes are given lower chances for involvement in routing decision whose energy levels are not sufficient. In addition, the multi-criteria for the selection of next-hops improves the forwarding process consistency.

**Fig 17:**
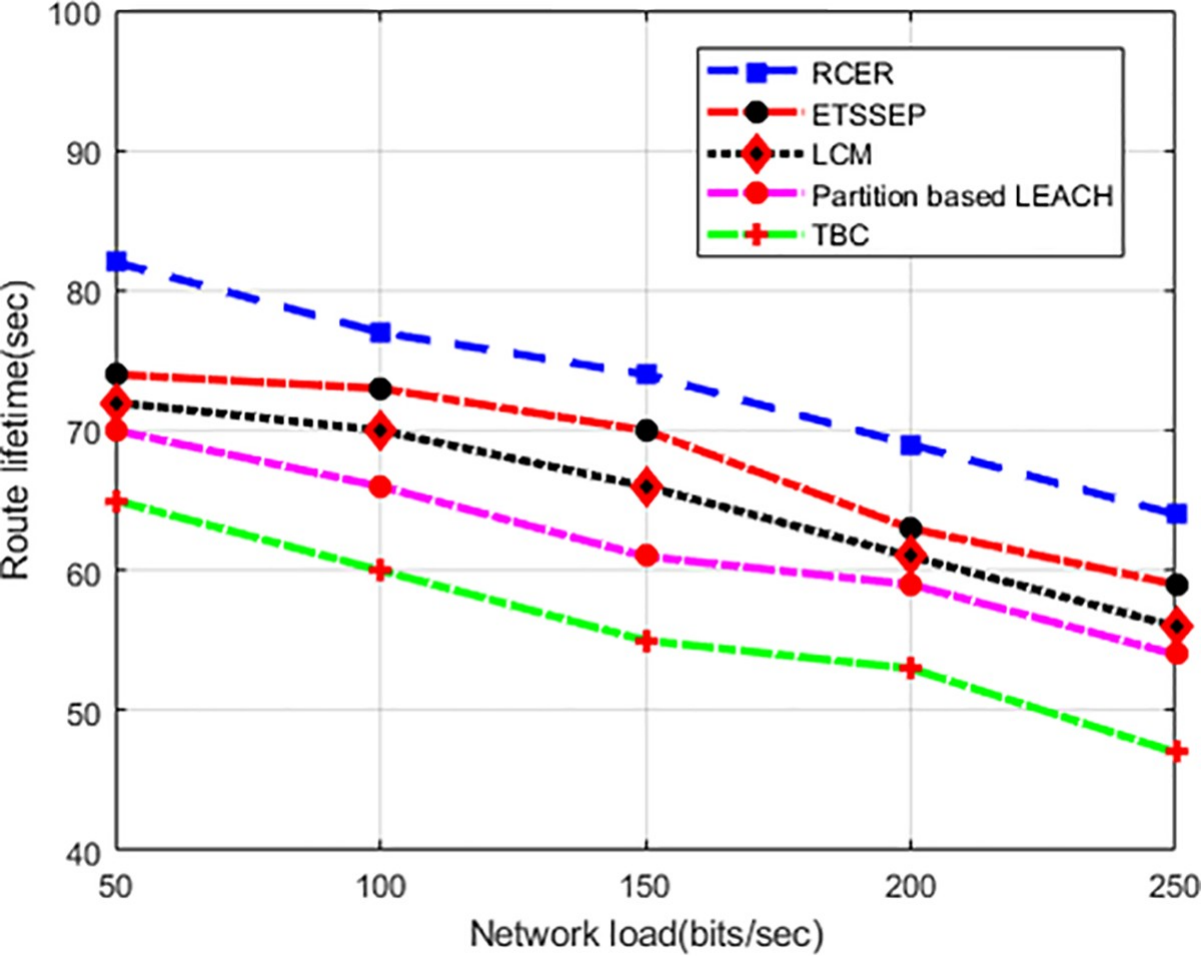
Route lifetime in varying network load.

“Fig 17” depicts the behavior of RCER than existing schemes in terms of route lifetime under varying network load. The simulation results show that RCER has superior performance, as it conquered 17%, 18%, 37%, and 43% improvement. In high network load scenarios, existing schemes reduced the route lifetime because of heavy network congestion and a number of re-transmissions. In addition, detection of exhausted and higher latency nodes on routing paths, RCER leads to robust routing with relatively stable performance.

#### 6.2.6. Packet delivery ratio analysis

**Fig 18:**
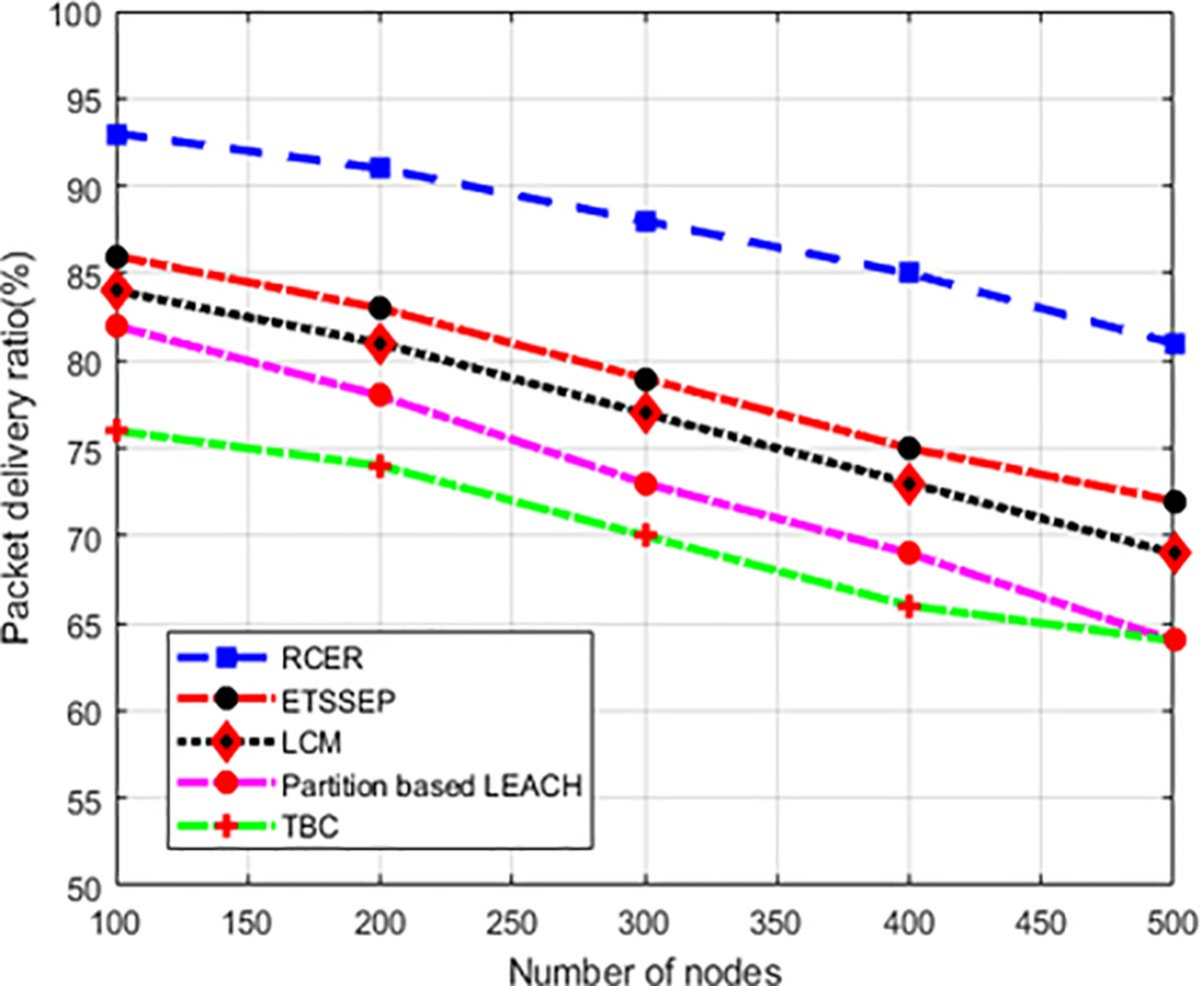
Packet delivery ratio in high-density nodes.

To evaluate the network reliability, “Fig 18” depicts the simulation results of RCER with other solution under a varying number of nodes scenario. Based on experimental results, it is seen that RCER protocol obtained high packet delivery ratio than an existing solution as 20%, 21%, 24%, and 37%. This is due to that unlike ETSSEP, TBC, LCM and Partition based LEACH schemes, the constructed data communication paths of RCER protocol are more reliable, as link quality factor is incorporated in routing decision. In addition, the energy de-efficient intermediate nodes are also avoiding from data routing, and based on the multi-criteria RCER protocol selects next-hop in reliable manner.

**Fig 19:**
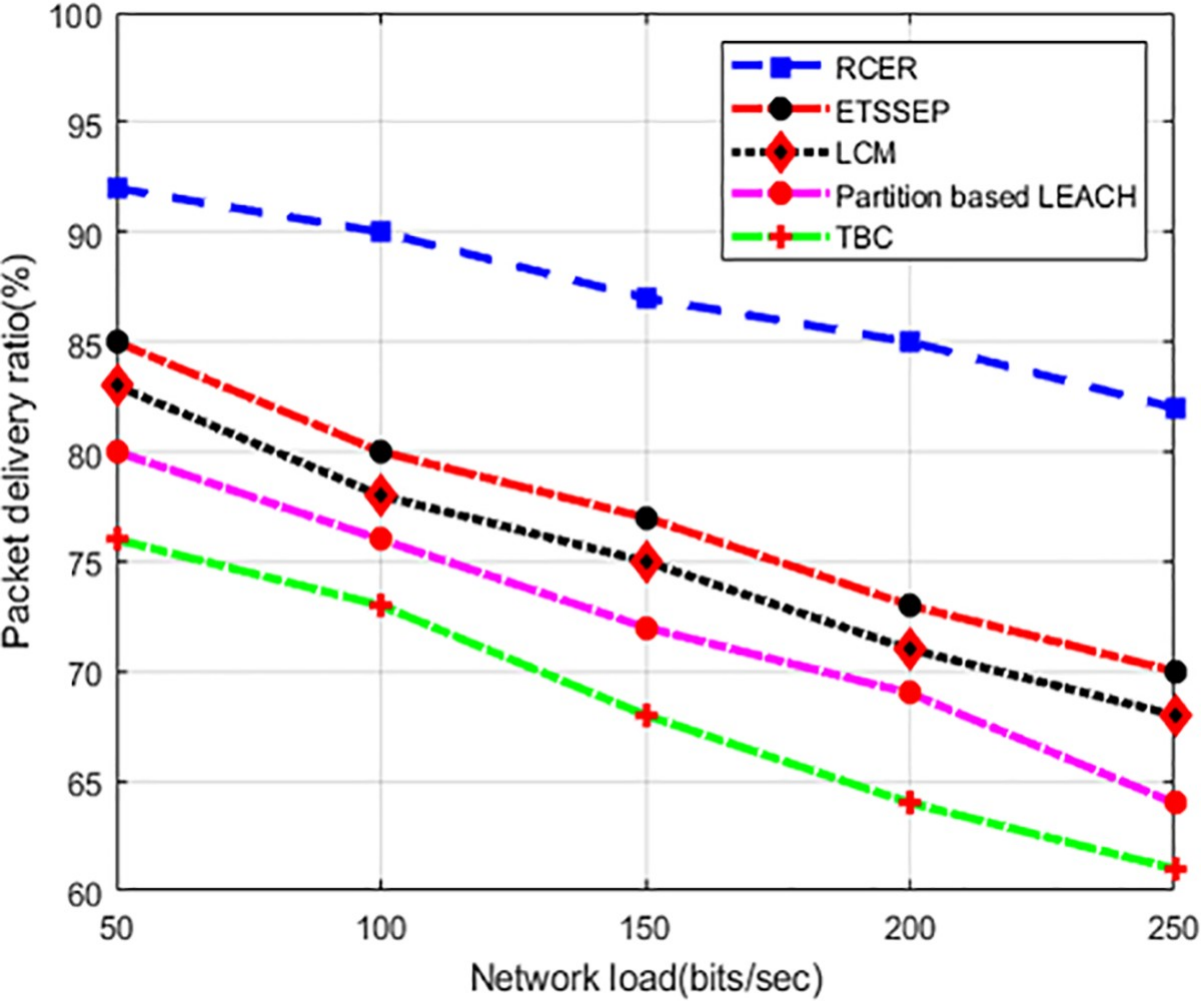
Packet delivery ratio in varying network load.

“Fig 19” illustrates the behavior of RCER in the comparison of existing schemes. The simulation experiments are performed to measure the reliability in terms if packet delivery ratio under varying network load. It is seen that RCER obtained better performance of reliability than existing schemes like 19%, 20%, 23%, and 31% because more priority is given to those next-hops that are optimum for data forwarding. In high network load, the existing schemes produce high network congestion because of the selection process of next-hop in non-optimal, as a result, the packet delivery ratio is low. Moreover, as RCER periodically identifies the link congestion and defective nodes on the basis of latency epoch, thus overburden nodes and links are given low weightage for data forwarding and accordingly, RCER protocol succeeded to minimize the data delivery interruption and improves overall network performance.

## 7. Conclusion

RCER protocol addresses the issue of energy efficiency and improved the routing performance of WSN within realistic network scenarios. However, most of the existing solution formed unbalanced clusters and use only distance factor in routing decision, which sources a lot of re-transmissions and transmission cost. Some existing schemes attempted to improve the selection of next-hop by using neighbor’s information. However, such schemes lack considering network measurements such as congestion on wireless links, which results in reducing data delivery performance with route instability. Basically, RCER proposed energy efficient heterogeneous cluster-based protocol with reliable routing. RCER constructs the geographical sized clusters and exploits light-weight multi-criteria for the selection of next-hop. Furthermore, forwarding data packets to overburden links are eliminated and positions of next-hops are restore based on nodes status. The simulation results expose the RCER protocol significantly improved energy consumption, network lifetime, network throughput, end-to-end delay and route lifetime in the comparison of existing schemes. In future work, we plan to measure the performance of RCER protocol on mobile sensors.
