# Correction: RCER: Reliable Cluster-based Energy-aware Routing protocol for heterogeneous Wireless Sensor Networks

**DOI:** 10.1371/journal.pone.0224319

**Published:** 2019-10-17

**Authors:** Khalid Haseeb, Naveed Abbas, Muhammad Qaiser Saleem, Osama E. Sheta, Khalid Awan, Naveed Islam, Waheed ur Rehman, Tabinda Salam

The third author’s name is spelled incorrectly. The correct name is: Muhammad Qaiser Saleem.

There is an error in affiliation 2 for author Muhammad Qaiser Saleem. The correct affiliation 2 is: College of Computer Science and Information Technology, Al Baha University, Al Baha, Saudi Arabia.
